# Diazepam nasal spray administration is effective to control seizure clusters irrespective of time of day

**DOI:** 10.3389/fneur.2024.1335421

**Published:** 2024-05-24

**Authors:** Kore Liow, James W. Wheless, David F. Cook, Adrian L. Rabinowicz, Enrique Carrazana

**Affiliations:** ^1^Hawaii Pacific Neuroscience, Honolulu, HI, United States; ^2^John A. Burns School of Medicine, University of Hawaii, Honolulu, HI, United States; ^3^Le Bonheur Children’s Hospital, University of Tennessee Health Science Center, Memphis, TN, United States; ^4^Neurelis, Inc., Honolulu, HI, United States; ^5^Center for Molecular Biology and Biotechnology (CMBB) in the Charles E. Schmidt College of Science at Florida Atlantic University, Boca Raton, FL, United States

**Keywords:** acute repetitive seizures, administration, intranasal, circadian, cycles, diazepam, epilepsy, rescue therapy

## Abstract

**Introduction:**

Neurologic circadian influences, including sleep/wake transitions, processes (e.g., hormonal variation), and behavioral patterns (e.g., consumption of food and oral medications), may affect seizure patterns. Specific circadian patterns of seizures have been reported depending on type, onset location, and severity; however, data on patterns for patients with seizure clusters and effectiveness of rescue therapy by time of day are limited.

**Methods:**

We conducted *post hoc* analyses using patient diary data from the phase 3 safety study of diazepam nasal spray, which is indicated for acute treatment of seizure clusters in patients with epilepsy aged ≥6 years. Patients were administered age- and weight-based doses; second doses could be administered if needed to control a seizure cluster. We assessed clock timing of seizure-cluster onset along with second-dose use as a proxy for effectiveness. Treatment-emergent adverse events were recorded.

**Results:**

Seizure-cluster onset was observed to be generally highest during mornings and late evenings and lowest in the early evening and middle of the night. Second-dose use was not consistently associated with a specific time of day. The safety profile was consistent with that expected from previous studies of diazepam nasal spray.

**Conclusion:**

These results suggest that diazepam nasal spray can be effectively administered at any time of day.

## 1 Introduction

Diurnal rhythms have been proposed to contribute to drug-resistance in some epilepsies (1). Processes that have circadian variation (e.g., hormones, body temperature, sleep, wakefulness) (1) and other factors that might be associated with daily behavioral patterns (e.g., effect of food or drug–drug interactions for daily antiseizure drugs) have not been adequately studied in relationship to control of epileptic seizures.

### 1.1 Circadian patterns of seizures

Specific circadian patterns and sleep/wake distributions of seizures have been reported, which may be associated with the seizure type, onset location, and severity, with implications for timing of treatment (2–4). One study showed that complex febrile seizures were more frequent during the day and evening than overnight, with severity stable for 24 h (5). Another study demonstrated diurnal differences in focal seizures based on the region of the brain; frontal lobe seizures occurred mostly during sleep, while temporal lobe seizures and occipitoparietal lobe seizures were more common when awake (6). Additionally, seizure severity has been shown to have circadian and longer time scale fluctuations specific to individual patients (7).

A study looking at Seizure Tracker database diary data found that seizure rates were highest early in the day (peak at 7 a.m.) and lowest overnight (after 6 p.m.) (8). Another study looking at continuous intracranial electroencephalography and seizure diaries in patients with focal epilepsies found 5 seizure peak times (12 a.m., 3 a.m., 9 a.m., 2 p.m., and 6 p.m.) (9). Additionally, interictal activity may be associated with a circadian pattern that is specific to the individual patient (10). In a study of the association between interictal epileptiform activity and circadian and multidien rhythms, variability was shown among patients with epilepsy; however, stability was shown for individual patients for many years (11).

### 1.2 Seizure cluster patterns

As with individual circadian seizures, seizure clusters also are characterized by patterns of occurrence (12). Seizure clusters (also known as acute repetitive seizures) are acute seizure emergencies involving intermittent increases in seizure activity that may occur despite treatment with antiseizure drugs (13). Currently, no definition of seizure clusters is widely accepted in the literature; operational definitions include a minimum number of seizures within a period of hours (e.g., ≥2 seizures during a 24-h period) (12). Untreated seizure clusters are associated with morbidity and mortality, including increased risk of status epilepticus and the likelihood of emergency room visits (14).

Although seizure clusters can occur at any time during the day, diurnal variation was reported from data examined in another study of the Seizure Tracker database (2). More severe seizure patterns, including seizure clusters, were most common in the daytime, and in general, seizure clusters occurred more frequently during the daytime than nighttime compared with isolated seizures. These data suggest that patients may have differing levels of risk during the day and at night that could be used to time therapy (2) or increase vigilance per individual risk.

### 1.3 Rescue therapy for seizure clusters

Regarding rescue therapy for seizure clusters, a goal of intranasal administration is to circumvent gastrointestinal metabolism, thus reducing the potential for associated variability and delay in drug absorption (15, 16). Dose volume and components of an intranasal formulation, including nasal-mucosal absorption enhancers, are crucial differentiators in the evaluation of nasal-specific antiseizure drugs.

There are few time-of-day data specifically for patients with seizure clusters. Here, we examine data from patients with epilepsy experiencing seizure clusters who were enrolled in the phase 3 safety study of diazepam nasal spray. This *post hoc* time-of-day analysis of onset of treated seizure clusters evaluated possible clinically relevant changes in effectiveness, as measured by second-dose usage. Diazepam nasal spray is approved by the US Food and Drug Administration (FDA) for acute treatment of intermittent, stereotypic episodes of frequent seizure activity (i.e., seizure clusters, acute repetitive seizures) that are distinct from a patient’s usual seizure pattern in patients aged ≥6 years (17). This intranasal formulation includes an alkylsaccharide absorption–enhancing excipient and is delivered in a small volume (100 μL solution per nostril, which is less than the total volume of the nasal cavity) [~200 μL] (15). Diazepam nasal spray has demonstrated a single peak concentration, suggestive of a single absorption pathway through the nasal mucosa (18, 19).

## 2 Methods

### 2.1 Study design and treatment

A full description of the methods for the long-term phase 3 real-world-use safety study of diazepam nasal spray has been published (20). A diagnosis of focal or generalized epilepsy with motor seizures or seizures with a clear alteration of awareness was required; however, patients’ specific diagnoses were not prospectively captured.

Patients aged 6–65 years received 5, 10, 15, or 20 mg of diazepam nasal spray (based on age and weight) as acute treatment for seizure clusters. Patients and care partners were instructed that second doses could be administered 4–12 h after the initial dose if needed to control a seizure cluster; doses could be adjusted by the investigator, including the timing of second doses (21), according to the investigator’s judgment and perceived clinical need if necessary for effectiveness and safety (20). Thus, data existed for second doses administered by patients or care partners prior to 4 h after the initial dose, and an analysis of these data has been published (22). Seizure timing and drug administration were recorded in patient diaries. Incidence and severity of overall treatment-emergent adverse events (TEAEs) were recorded, and relationship to therapy was assessed (20). Data were summarized with descriptive statistics.

### 2.2 Analyses of time of day of seizure clusters

For this study, seizure clusters were operationally defined as ≥2 seizures during a 24-h period (21). The 12-h periods used to record onset of treated seizure clusters were 8 a.m.–8 p.m. (i.e., the daytime period) and 8 p.m.–8 a.m. (i.e., the nighttime period). Timing was further broken into six 4-h clock periods: 12 a.m.–4 a.m., 4 a.m.–8 a.m., 8 a.m.–12 p.m., 12 p.m.–4 p.m., 4 p.m.–8 p.m., and 8 p.m.–12 a.m. Use of second doses was assessed within 24 h of the initial dose (21).

### 2.3 Assessment of second-dose use

Second-dose use as a proxy for effectiveness (i.e., to determine the proportion of seizure clusters for which a second dose was administered to control a seizure cluster) was examined using the 12- and 4-h time periods. The number of treated seizure clusters for which a second dose was used was grouped by the clock time of the initial dose. All second doses and the second doses administered within 4 h of the initial dose were assessed.

Although subjects had multiple seizure clusters, it was assumed that seizure clusters were independent observations. Thus, McNemar’s test was used for analysis of differences between second dose use during the day and at night.

## 3 Results

### 3.1 Overall effectiveness and safety

The full overall effectiveness and safety results of the phase 3 safety study are published (20). A total of 4,390 doses of diazepam nasal spray were administered for a total of 3,853 reported seizure clusters among the 163 patients in the safety population (Table 1). Second doses were reported for 12.6% of seizure clusters (485/3,853) (20).

**Table 1:** Demographic characteristics and treatment exposure (safety population, *N* = 163).

**Variable**	**Value**
**Sex, *n* (%)**	
Male	74 (45.4)
Female	89 (54.6)
**Age, y**	
Mean (SD)	23.1 (15.1)
6–17 y, *n* (%)	78 (47.9)
≥18 y, *n* (%)	85 (52.1)
**No. of second doses, *n* (%)**	
0	84 (51.5)
1	33 (20.2)
2	10 (6.1)
3–5	15 (9.2)
>5	21 (12.9)
**Duration of exposure, *n* (%)**	
<6 months	9 (5.5)
6 to <12 months	21 (12.9)
≥12 months	133 (81.6)

In the overall study safety profile, 134 patients (82.2%) had at ≥1 TEAE, 50 (30.7%) had serious TEAEs, and 30 (18.4%) had TEAEs that were potentially treatment related (20). No serious TEAEs or discontinuations were considered to be related to treatment, and there were no cases of respiratory depression. One death occurred (sudden unexpected death in epilepsy) that was deemed unlikely to be related to treatment (20).

Forty-four patients (27.0% of the safety population) reported serious TEAEs requiring/prolonging hospitalization. The most common of these were seizure (14.7%), pneumonia (4.3%), and status epilepticus (4.3%). Six study drug–treated seizure clusters (0.16% of 3,853 seizure clusters) progressed to status epilepticus, and all resulted in hospitalization.

### 3.2 Onset of seizure clusters by time of day

**Figure 1:**
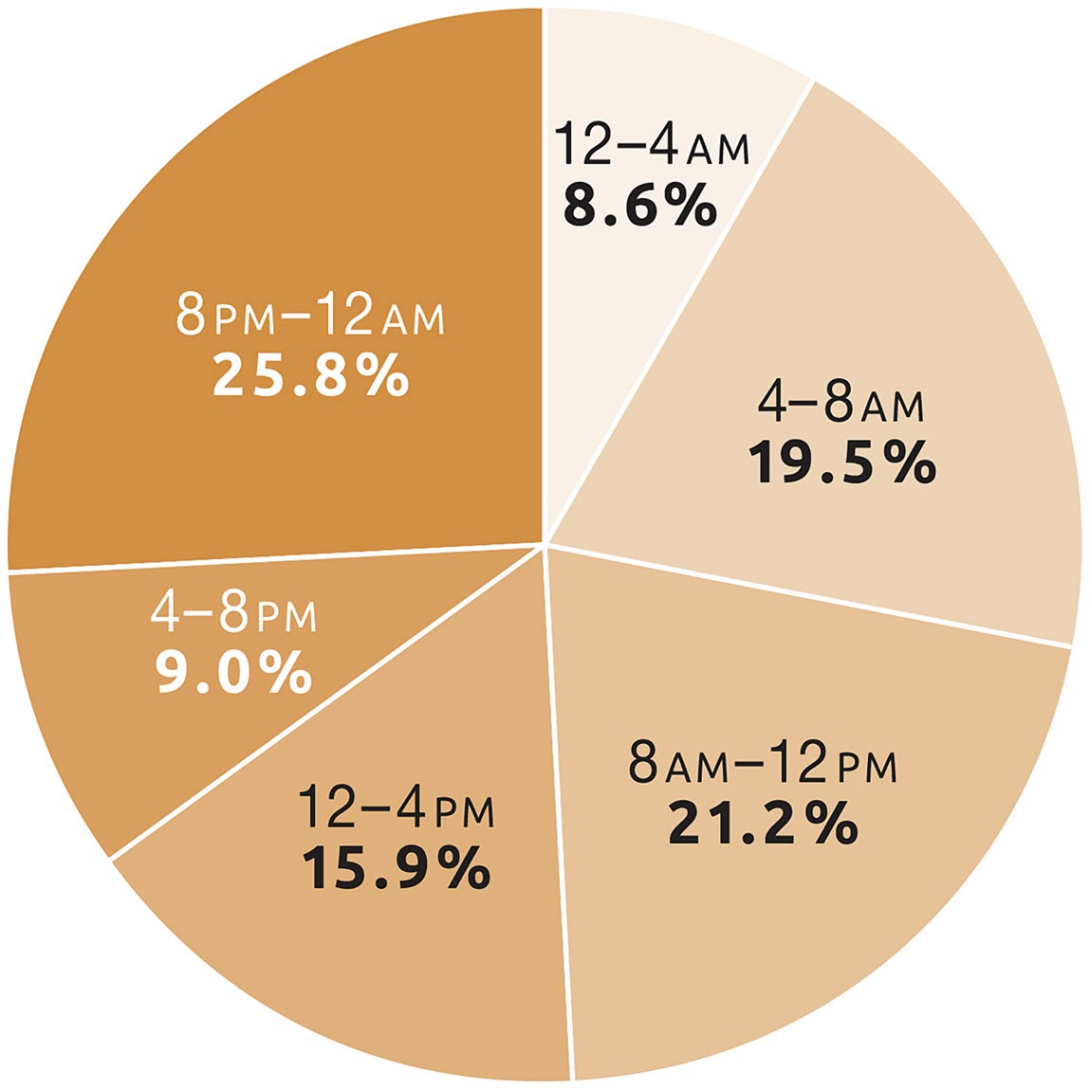
Percentage of seizure clusters by time of day of seizure occurrence (total = 3,853; safety population, *N* = 163).

Start times were reported for all 3,853 seizure clusters, with 1,774 (46.0%) between 8 a.m. and 8 p.m. (day) and 2,079 (54.0%) between 8 p.m. and 8 a.m. (night). The pattern of onset of seizure clusters (i.e., administration of initial dose) is shown in Figure 1. Onset was generally highest during mornings and late evenings and generally lowest in the afternoon, early evening, and middle of the night.

### 3.3 Usage of second doses by time of day

**Figure 2:**
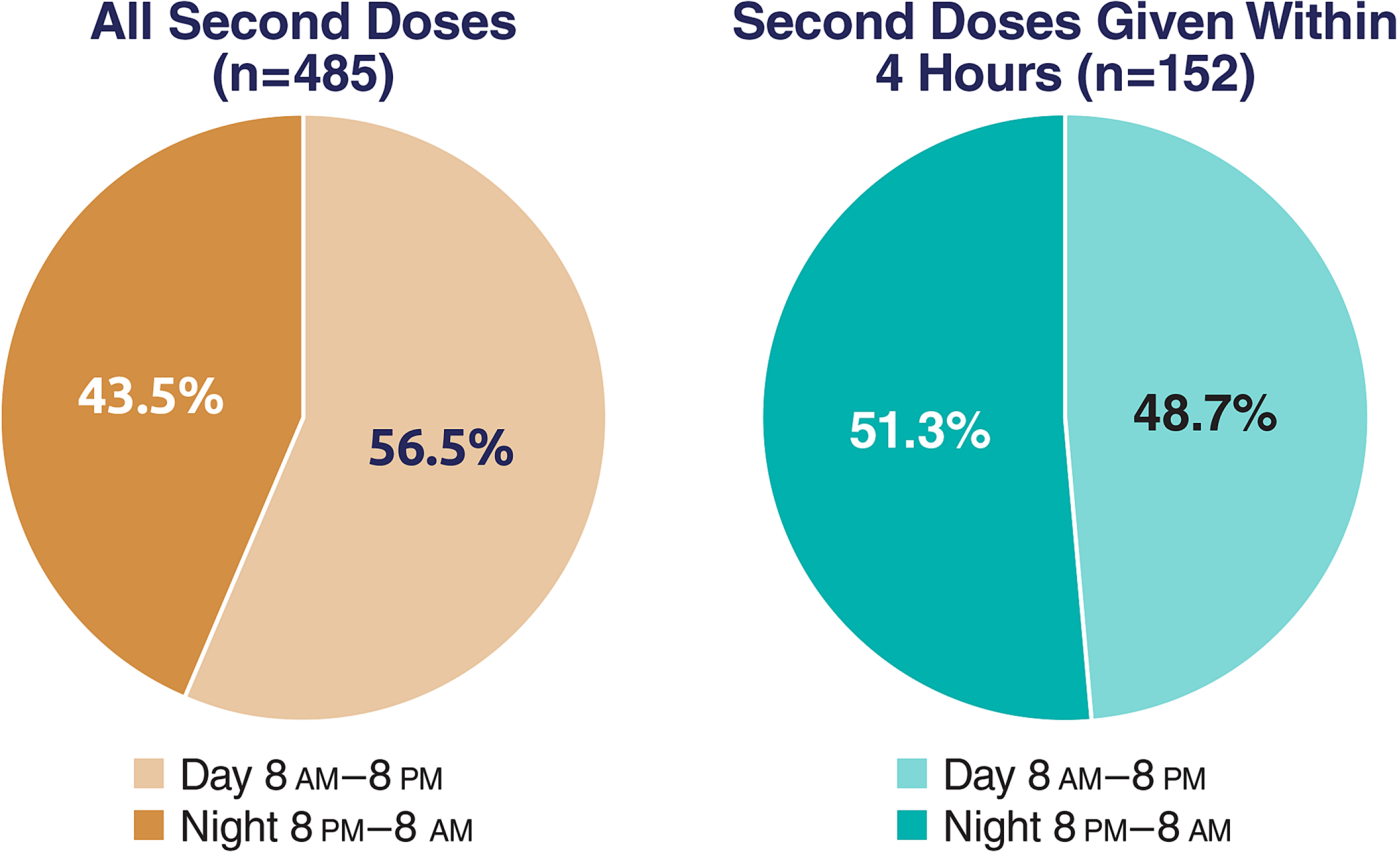
Proportion of all second doses during daytime or nighttime.

When evaluated by 12-h periods, of all second doses administered within 24 h of the first (*N* = 485), slightly more were used during the day (8 a.m.–8 p.m.) than at night (i.e., 56.5% vs. 43.5%). Using McNemar’s test, the difference between day and night was statistically significant (*p* = 0.004). Usage of second doses within 4 h of the initial dose was similar between day and night (48.7% vs. 51.3%), and the difference was not significant (*p* = 0.75; Figure 2).

**Figure 3:**
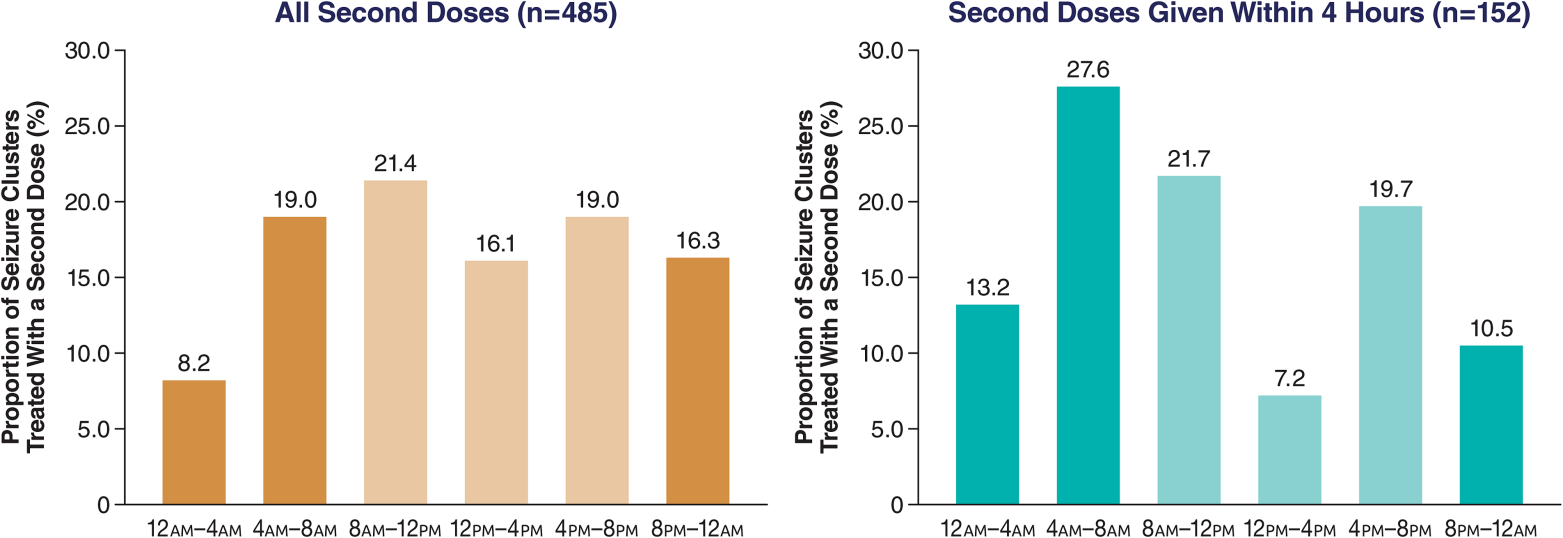
Proportion of all second doses of diazepam nasal spray based on time of day of first dose (4-h periods).

The 4-h periods across 24 h showed no apparent trends for either day or night. Using 4-h periods, use of second doses within 4 or 24 h of the first dose was notably lower around 12 p.m.–4 p.m. compared with adjacent daytime periods (Figure 3).

## 4 Discussion

The results from these *post hoc* analyses of data from a long-term safety study evaluating diazepam nasal spray suggest a circadian pattern of seizure clusters, which has implications for monitoring and treatment of patients. Seizure severity was not captured in the study’s patient diaries; however, seizure clusters have been defined as severe seizures (2). Serious TEAEs of status epilepticus or leading to hospitalization were rare; therefore, no diurnal pattern could be established. None of these events were deemed related to treatment.

In this study, a modest association was shown between time of day of administration and effectiveness based on second-dose usage in 24 h for controlling seizure clusters, suggesting that the drug can be used effectively at any time (e.g., throughout the day, throughout the ictal cycle). Also, no trends in effectiveness were shown by the number of second doses administered within 4 h of the first dose, indicating no observed effect on onset of effectiveness. As no effect on second-dose use within 4 h was observed during the daytime, this suggests a lack of clinically relevant food effect. This is as expected with intranasal administration that bypasses the gastrointestinal tract, and the administered formulation contains the absorption enhancer dodecyl maltoside (23).

Also, in the overall long-term safety study, 3,853 treated seizure clusters were reported, with second doses used for 485 seizure clusters (12.6%) (20). In a review of second-dose use in the large studies of the 3 medications with FDA approval for seizure clusters (i.e., rectal diazepam, intranasal midazolam, diazepam nasal spray), the need for a second dose was assessed at 6, 12, and/or 24 h, as timing varied in the noncomparative long-term studies evaluating these medications (24) In these studies, only one dose was needed (i.e., no second dose was administered) for 77.0% of seizure clusters across 12 h with diazepam rectal gel, for 61.5% of seizure clusters across 6 h with intranasal midazolam, and for 94.2%, 91.7%, and 87.4% of seizure clusters across 6, 12, and 24 h, respectively, with diazepam nasal spray, supporting the effectiveness of diazepam nasal spray (24). Among the patients using the most second doses in the intranasal diazepam study, 50% of the second doses were concentrated in 7 patients (241 of 485 doses), suggesting that second-dose use is more closely related to the specific patient than to when a dose was administered. In all 79 patients using second doses, only 1 second dose was used by 33 (42%) patients during the study, and the use of second doses was consistently low across 24 h (21).

### 4.1 Limitations

Limitations of the diazepam nasal spray phase 3 study have been previously published (20). The underlying etiology of patients’ epilepsy was not examined as a variable in this analysis. Use of patient diaries may be limited by understanding of instructions, reporting completeness, and seizure awareness (25). Also, the availability of the care partners for reporting data during working or sleeping hours may have been a limitation. Further, the *post hoc* nature of these analyses is a limitation, affecting the potential applicability of any statistical comparisons. However, the study may be valuable to detect patterns or trends by comparing subgroups that were not directly examined in the study.

In conclusion, the findings of these *post hoc* analyses indicate no clear association between time of day of seizure clusters and administration of second doses of diazepam nasal spray for seizure clusters in the 4 h following the initial dose, and safety was not shown to be affected. Second-dose use, a proxy for treatment effectiveness, had a small difference between day and night over 24 h, suggesting diazepam nasal spray can be administered at any time of day with an expectation of consistent effectiveness. Further analysis of seizure patterns is warranted.

## Data availability statement

The original contributions presented in the study are included in the article/Supplementary material, further inquiries can be directed to the corresponding author.

## Ethics statement

The study from which the data used in these post hoc analyses were approved by the ethics committees or institutional review boards at each site (a full list is available as Supplementary material). The study was conducted in accordance with the ethical principles originating from the Declaration of Helsinki and consistent with good clinical practice and applicable regulatory requirements. Written informed consent for participation in the study was provided by patients or parents/guardians.

## Author contributions

KL: Investigation, Methodology, Visualization, Writing – review & editing. JW: Investigation, Methodology, Visualization, Writing – review & editing. DC: Methodology, Visualization, Writing – review & editing. AR: Conceptualization, Methodology, Supervision, Visualization, Writing – review & editing. EC: Conceptualization, Methodology, Supervision, Visualization, Writing – review & editing.
