# Supplementary material for: Diazepam nasal spray administration is effective to control seizure clusters irrespective of time of day
Source: Front Neurol. 2024 May 24;15:1335421. doi: 10.3389/fneur.2024.1335421 (PMC11157958; doi:10.3389/fneur.2024.1335421)
Supplement: Supplementary file 1 [file Table_1.docx]

# Diazepam Nasal Spray Administration Is Effective to Control Seizure Clusters Irrespective of Time of Day

Kore Liow, James W. Wheless, David F. Cook, Adrian L. Rabinowicz, Enrique Carrazana

Ethics committees/Institutional review boards providing approval of study procedures

**Brany Institutional Review Board**

1981 Marcus Ave. Ste 210

Lake Success, NY 11042

**Children’s Hospital of Orange County Industry Track**

Institutional Review Board

1201 W. La Veta Avenue

Orange, CA 92868

**Icahn School of Medicine at Mount Sinai**

Institutional Review Board

One Gustave L. Levy Place Box 1081

345 E 102nd St., Ste 200

New York, NY 10029

**Mayo Clinic Institutional Review Board**

200 First St SW

Rochester, MN 55905

**Providence Health Services**

Institutional Review Board

Providence Portland Medical Center

5251 NE Glisan, Building A, 3rd Floor

Portland, OR 97213

**The Children’s Hospital of Philadelphia**

Institutional Review Board

2716 South Street

4th Floor

Philadelphia, PA 19104

**University of Tennessee**

Health Science Center

Institutional Review Board

910 Madison Ave., Suite 600

Memphis, TN 38163

**University of Virginia**

Institutional Review Board for Health Sciences Research

PO Box 800483

University of Virginia

Charlottesville, VA 22908

**USC Children’s Hospital of Los Angeles**

4650 Sunset Blvd., #23

Los Angeles, CA 920027

**Washington University in St. Louis**

Human Research Protection Office

Barnes Jewish Hospital

St. Louis Children’s Hospital

Washington University

660 South Euclid Ave.,

Campus Box 8089

St. Louis, MO 63110

**Western Institutional Review Board**

1019 39th Ave., SE, Suite 120

Puyallup, WA 98374-2115
